# Employment changes among Chinese family caregivers of long-term cancer survivors

**DOI:** 10.1186/s12889-020-09922-9

**Published:** 2020-11-25

**Authors:** Siqi Liu, Mingzhu Su, Nengliang Yao, Nan Zhang, Jialin Wang, Roger T. Anderson, Xiaojie Sun

**Affiliations:** 1 Centre for Health Management and Policy Research, School of Public Health, Cheeloo College of Medicine, Shandong University, Jinan, No.44 Wenhuaxi Road, Jinan, 250012 Shandong China; 2 NHC Key Lab of Health Economics and Policy Research (Shandong University), No.44 Wenhuaxi Road, Jinan, 250012 Shandong China; 3 Shandong Cancer Hospital and Institute, Shandong First Medical University and Shandong Academy of Medical Sciences, No.440 Jiyan Road, Jinan, 250117 Shandong China; 4 Department of Public Health Sciences, University of Virginia, 200 Jeanette Lancaster Way, Charlottesville, VA 22903 USA

**Keywords:** Family caregivers (FCGs), Cancer survivors, Employment changes, Cancer-related characteristics, In post-treatment phase

## Abstract

**Background:**

Family caregivers (FCGs) play a key role in the plan of care provision for long-term cancer survivors, yet few studies have been conducted on the impact of long-term caregiving on FCGs and their employment patterns. This study aims to further our understanding of the effect that caregiving role has on FCGs by identifying what cancer-related characteristics influence reduction of employment hours among FCGs in the post-treatment phase in China.

**Methods:**

A total of 1155 cancer survivors participated in this study. Patients reported changes in the employment patterns of their FCGs. Descriptive analysis looked at demographic and cancer-related characteristics of cancer survivors and types of FCGs’ employment changes in both primary- and post-treatment phases. Chi-square test was used to statistically test the association between survivors’ characteristics and changes in FCGs’ hours of labor force work in post-treatment phase. Separate multivariable logistic regression models were used to examine the relationship between cancer-related characteristics of participants and employment reduction patterns among FCGs in post-treatment phase while controlling for demographic factors.

**Results:**

In the primary-treatment phase, 45.6% of all FCGs reduced their working hours and 17.4% stopped working altogether. In the post-treatment phase, 25.2% of FCGs worked fewer hours and 6.6% left the workforce completely. The results show that a higher probability of change in employment hours among FCGs is associated with the following patient characteristics: having comorbidities, receiving chemotherapy treatment, limited ability to perform physical tasks, limited ability to perform mental tasks, and diagnosis of stage II of cancer.

**Conclusions:**

Care for cancer patients in both primary- and post- treatment phases may have substantial impacts on hours of formal employment of Chinese FCGs. Interventions helping FCGs balance caregiving duties with labor force work are warranted.

**Supplementary Information:**

The online version contains supplementary material available at 10.1186/s12889-020-09922-9.

## Background

Data presented by GLOBOCAN 2018 indicated that there would be an estimated 18.1 million new cancer cases all over the world in 2018 with approximately 24% of them residing in China [1]. This is largely due to population growth and increase in longevity, but another contributing factor is the change in disease patterns in China. China sees alarming increase in cancer incidence accompanied by the rise in survival rates of cancer patients due to diagnosis and treatment advances [2, 3]. The National Central Cancer Registry of China (NCCR) reported that among all cancer types in China, the age-standardized 5-year survival rate rose from 30.9% during the period between 2003 and 2005 to 40.5% a decade later (2012–2015) [4]. As a result, initiatives to support cancer survivors and their families have become imperative.

Throughout the course of illness - from the time of diagnosis to the time of death or recovery - cancer continues to undermine the well-being of cancer patients and their families. Even cancer survivors, who have completed the treatment, may experience cancer after-effects [5]. For example, the lasting outcomes of cancer include fear of disease recurrence, difficulties in adjusting and adapting to one’s new lifestyle, problems in social relationships with others, confusion about the survivorship period and the need for external assistance [6]. Continuous care from family members plays a crucial role in the process of effective long-term care, especially during the post-treatment period after patients’ return to their communities. The term “family caregivers (FCGs)” generally refers to individuals (e.g. adult children, spouses, parents, and siblings) who offer informal domestic care for patients. FCGs work without external compensation and might need to spend months or even years of time and energy on care and support for patients. Such caregiving demands may bring potentially negative impacts on physical, emotional, social or economic well-being of the caregiver [7].

Extensive research has shown that FCGs caring for cancer survivors bring positive influence on multiple aspects of patient care such as assisting further rehabilitation of patients, relieving household disease burden, contributing to the development of community long-term care, and lowering caregiving related costs for national healthcare system [8–10]. However, these benefits may sometimes come at the cost of hardship for the FCGs. Firstly, unlike full-time professional caregivers, for whom caregiving is usually their main employment and source of income, FCGs have to reconcile the role of family care provider with their formal employment. This “double” role of care provider and breadwinner puts additional pressures on FCGs and has significant financial implications for caregivers and their families. Further, by contrast with many other chronic diseases, cancer often causes sharp health deterioration within a relatively short period, which puts significant pressure on FCGs as they have to spend many hours a day offering intensive care [7, 11, 12]. Furthermore, cancer patients typically experience more complicated and malignant symptoms than do patients with other chronic diseases due to painful treatment methods and the nature of the disease. This compels their FCGs to spend a significant amount of time and effort on providing a wide range of care while constantly searching for more ways to improve patients’ health conditions [13]. Moreover, the specificity of illness trajectory of cancer (i.e. diagnosis, treatment, transition off treatment, survivorship, reoccurrence, secondary progression and end-of-life) can also be a source of additional stress for cancer patients and their families [14]. Cancer reoccurrence, for example, can unexpectedly increase the burden of care provision on FCGs. Individuals may have no evidence or symptom of the clinical course, and it might only be diagnosed again after several years. Given all these and other challenges associated with cancer care, it’s not surprising that FCGs often try to reduce or even give up employment completely.

Research showing that caring for cancer patients interferes with formal employment of FCGs is mainly concentrated in western countries. A Canadian study, for example, showed that more than 77% of FCGs missed work in the terminal period of cancer care [15]. Another study in the US estimated that 22% of FCGs decided to reduce their working time or even leave their jobs to provide care for the patients [16]. A more recent research in the US showed that nearly 25% of cancer survivors stated that their FCGs had to make working time accommodations because of providing care, and 8% of them had FCGs who had to take time off from work for more than 2 months [17]. Also, a study conducted in Canada showed that, compared to their colleagues who have no care responsibilities, FCGs demonstrated lower working productivity, because they were more exhausted; required more days off from work; and left the office earlier due to caregiving demands. In addition, FCGs are more likely to give up career advancements and opportunities for promotion, and may even leave their formal job to fully concentrate on their caregiving role [18].

In China, owing to its cultural context, most Chinese cancer survivors mainly rely on their family members for daily life support instead of hiring professionals [19–22]. Yet, to our best knowledge, there are still very few studies examining employment consequences for cancer FCGs in China. Research on Chinese FCGs mostly focuses on such factors as caregiving burden, caregiving activities, health-related quality of life (HRQoL), or mental health. For example, a study has shown that caregiving burden on Chinese FCGs caring for lung cancer patients is associated with patient age, type of health insurance, disclosure of the diagnosis to patients and the social support received by the caregiver [21]. Meanwhile, the type and amount of information disclosed to patients and FCGs was not found to be of particular importance [20]. Previous research also showed that offering informal care to family members may drastically reduce the quality of life of FCGs, and especially their mental health [23]. A survey of Chinese Canadians has indicated that caregiving activities affect employment and economic situation in the family [24], but these findings did not stimulate further research on this topic. It is still unclear what the specific employment effects of cancer caregiving are and how to identify high-risk groups of FCGs or differentiate between their level of vulnerability to employment-related challenges caused by caregiving activities. In addition, under the household registration system (Hukou) that separates rural residents and urban residents in modern day China, rural residents tend to face more occupational segregation and earnings inequality when compared with urban employees [25]. Long-term cancer care puts a great number of Chinese FCGs at a higher risk of reducing or quitting work altogether, thus further aggravating employment-related challenges that Chinese people face. Hence, a better understanding of cancer-related characteristics of patients and their relationship to employment changes among cancer FCGs could provide a fresh view into societal and economic implications of cancer caregiving in China. This, in turn, will provide policy relevant evidence for targeting interventions and help FCGs at risk of reduced or lost employment.

To sum up, this study aims to 1) examine the effect of long-term caregiving on the employment status of FCGs caring for cancer patients; and 2) identify which cancer-related characteristics are associated with negative employment changes among FCGs in post-treatment phase.

## Methods

### Study design and participants

We used the Cancer Supplement of the Medical Expenditure Panel Survey (MEPS) as a template to design the survey for this study - “Your Experiences with Cancer in China” (Additional file 1 provided the related items from the Questionnaire of the survey). The survey was distributed to cancer patients sampled from official cancer registry system [26, 27]. The final in-person survey was conducted between March 2015 and March 2016. Stratified random sampling technique was used in this study. Eight county-level units across the western, central, and eastern regions of Shandong province were covered to obtain a representative sample of Shandong province in terms of demographic context and socioeconomic development. The sample was stratified by rural versus urban status. The final sample consisted of cancer survivors from 5 rural counties and 3 urban districts (details of geographic distribution of sampling regions are provided in Additional file 2). It is important to note that counties and districts are at the same level in the Chinese administrative division system. At the time of our survey, patients in rural counties were covered for the most part by the rural new cooperative medical scheme (RNCMS), and the majority of patients in urban districts enjoyed the urban employee basic medical insurance (UEBMI) or the urban resident basic medical insurance (URBMI).

For the purpose of this study, “cancer survivors” are defined as the patients who lived at least past one-year survival mark after completing the treatment. One thousand six-hundred cancer survivors who were diagnosed with the most common cancers (breast, lung, colorectal, and stomach) were randomly extracted from the local cancer registry system. At the beginning of the survey, all patients received an explanation about the purpose of the study and were asked to sign consent forms indicating willingness to participate in the study. Patients willing to participate in the study were interviewed face-to-face by trained investigators at their homes. Eligibility criteria for study participants were the following: 1) history of diagnosis with the most common cancers (lung, stomach, colorectal, breast) between 2011 and 2014; 2) at least 18 years old at the time of cancer diagnosis; 3) completion of primary cancer treatments (surgery, chemotherapy, and radiation therapy) at least 1 year ago; and 4) receiving care from FCGs who were employed at the time of diagnosis. In accordance with the above inclusion criteria, 445 participants were excluded, leaving 1155 participants for the analysis in the study.

### Study measures

Demographic characteristics of participants included residence area, age, gender, marital status, educational level, and annual household income; cancer-related characteristics of participants included comorbidity (yes/no), cancer site (breast/lung/stomach/colorectal), cancer stage at diagnosis (0-I/II/III-IV), type of treatment (chemotherapy/surgery/radiation therapy), limited ability to perform physical tasks (yes/no), limited ability to perform mental tasks (yes/no), and duration of disease since diagnosis (2-3 years/4-5 years). The variable “comorbidity” was identified as “yes” if any additional chronic disease (e.g. diabetes, cardiovascular diseases, respiratory diseases) was diagnosed.

For the purpose of this study, the “primary-treatment phase” was defined as the period during which patients have completed cancer treatment (i.e. chemotherapy, radiation, and/or surgery) within one year or are still undergoing the treatment in a hospital; the “post-treatment phase” was defined as the stage after the patients have stopped receiving the treatment for at least one year. Participating cancer survivors were asked the following two questions for evaluating employment changes of their FCGs: a) “Throughout your cancer treatment, did any of your family caregivers shorten their paid working hours because of your cancer diagnosis, its treatment or the after effects of the treatment?”; b) “After having completed your treatment for a period of at least one year, did any of your family caregivers shorten their paid working hours because of your diagnosis, its treatment or the lasting effects of the treatment?” The responses were categorized as follows: a) Increased working hours; b) Kept the regular working hours; c) Reduced working hours by less than half; d) Reduced working hours by half; e) Reduced working hours by more than half; f) Stopped working; g) Not applicable (retired or unemployed when they were first diagnosed).

Ethical approval for this study was obtained from the Ethics Committee of the School of Public Health at Shandong University (NO.20140201).

### Statistical analyses

Descriptive statistics were used to summarize sociodemographic and cancer-related characteristics of participants, as well as the employment status of the FCGs in both the primary- and post- treatment phases. Frequencies and percentages were applied for categorical variables. The analysis was a two-stage process and focused on a dichotomous outcome measure - Sustained Employment vs. Reduced Employment in post-treatment phase. Response item “b” was included in the “Sustained Employment” category, while the response items “c”, “d”, “e”, and “f” were included in the “Reduced Employment” category. A chi-square test with degrees of freedom determined by the respective categories of the variables in each row and column was conducted to test the significance of relationship between each variable and long-term employment reduction among FCGs (0 = Sustained; 1 = Reduced). All variables were included in the following separate multivariable logistic regression models to estimate the effect of cancer-related characteristics of patients on FCGs’ employment change (0 = Sustained; 1 = Reduced). Model 1 was conducted to preliminarily control for all demographic characteristics, and then models 2–10 were conducted for each of cancer-related characteristics, individually. All multivariable models were adjusted to control for the confounding factors of age (which was entered as a continuous variable), residence area (1 = Urban; 2 = Rural), gender (1 = Male; 2 = Female), marital status (1 = Others; 2 = Married), educational level (1 = Uneducated; 2 = Elementary school; 3 = Middle school; 4 = High school and above), and annual household income (1 = Less than 5000 CNY; 2 = 5000–19,999 CNY; 3 = 20,000–50,000 CNY; 4 = More than 50,000 CNY). The multivariable model for cancer site was adjusted for controlling age, residence area, marital status, educational level and annual household income, due to the inclusion of patients with breast cancer. Odds ratios and βs (95% confidence intervals) were reported. In this study, a two-tailed probability value of below 0.05 was considered statistically significant. All analyses were conducted using the SPSS 21.0 statistical package (SPSS Inc., Chicago, IL, USA).

## Results

### Description of characteristics of cancer survivors

As presented in Table 1, among 1155 cancer survivors included in this study, only a small fraction of participants (6.8%) reported that they were diagnosed at the stage III-IV of cancer; treatment types received by participants included chemotherapy (68.7%), radiation therapy (11.6%), and surgery (88.9%); nearly a quarter of participants (26.9%) were diagnosed with multiple chronic diseases; more than half of participants (55.3%) in the survey stated that their ability to perform physical tasks was limited, and approximately one-third of participants (31.3%) reported that their ability to perform mental tasks was limited.

**Table 1:** Characteristics of cancer survivors (*N* = 1155)

Characteristics	*N* (%)
Residence area	
Urban	379 (32.8)
Rural	776 (67.2)
Age group (years)	
≤ 40	45 (3.9)
40–55	299 (25.9)
56–65	384 (33.2)
> 65	427 (37.0)
Gender	
Male	496 (42.9)
Female	659 (57.1)
Marital status	
Married	1037 (89.8)
Others	118 (10.2)
Educational level	
Uneducated	185 (16.0)
Elementary school	403 (34.9)
Middle school	377 (32.6)
High school and above	190 (16.5)
Annual household income^a^, Chinese Yuan	
< 5000	182 (15.8)
5000-20,000	417 (36.1)
20,000-50,000	376 (32.6)
> 50,000	172 (14.9)
Missing	8 (0.7)
Cancer site	
Breast	406 (35.2)
Lung	163 (14.1)
Stomach	284 (24.6)
Colorectal	302 (26.1)
Stage at diagnosis	
0- I	497 (43.0)
II	405 (35.1)
III-IV	78 (6.8)
Missing	175 (15.2)
Type of treatment^b^	
Chemotherapy	794 (68.7)
Surgery	1027 (88.9)
Radiation therapy	134 (11.6)
Comorbidity	
Yes	311 (26.9)
No	813 (70.4)
Missing	31 (2.7)
Limited ability to perform physical tasks	
Yes	639 (55.3)
No	123 (10.6)
Not applicable^c^	393 (34.0)
Limited ability to perform mental tasks	
Yes	362 (31.3)
No	425 (36.8)
Not applicable^c^	368 (31.9)
Duration of disease since diagnosis	
2–3 years	745 (64.5)
4–5 years	410 (35.5)

^a^ 10,000 Chinese Yuan ≈1541 US dollars as of December 31, 2015;

^b^ Categories are not mutually exclusive because most patients received a combination of treatments

^c^ Participants who were retired or unemployed by the time they were diagnosed with cancer can be identified as “Not applicable”

### Employment changes experienced by FCGs

**Fig. 1:**
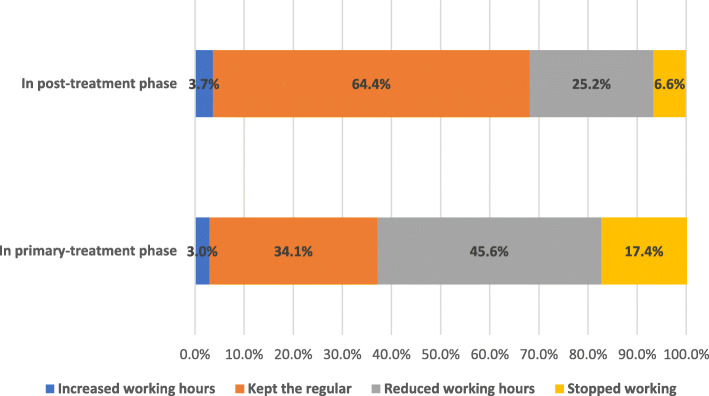
Proportions of different types of employment changes among FCGs

Figure 1 graphically represents proportions of different types of employment change among FCGs. In the primary-treatment phase, nearly half of FCGs (45.6%) reduced their working hours, and 17.4% of FCGs stopped working due to care provision responsibilities. In contrast, only a small percentage (3.0%) increased their working hours. In the post-treatment phase, nearly a quarter of FCGs (25.2%) chose to reduce their working hours to care for the patient, and a small number of FCGs (6.6%) stopped working. The difference between the employment changes of FCGs in the primary-treatment phase and the post-treatment phase is statistically significant (*P* < 0.01).

Table 2 summarizes the relationship between each patients’ characteristic and the FCGs’ employment time reduction in post-treatment phase. As can be seen, among the demographic variables, different residence area (*χ*^*2*^ *=* 52.542; *df* = 1; *P* < 0.001), gender (*χ*^*2*^ = 6.075; *df* = 1; *P* = 0.014), marital status (*χ*^*2*^ = 6.002; *df* = 1; *P* = 0.014), educational level (*χ*^*2*^ = 41.948; *df* = 3; *P* < 0.001), and annual household income (*χ*^*2*^ = 22.662; *df* = 3; *P* < 0.001) have statistically significant relationship with reduction in hours of labor force work among FCGs. As for cancer-related characteristics, comorbidity (*χ*^*2*^ = 8.356; *df* = 1; *P* = 0.004), chemotherapy treatment (*χ*^*2*^ = 10.205; *df* = 1; *P* = 0.001), limited ability to perform physical tasks (*χ*^*2*^ = 49.151; *df* = 1; *P* < 0.001), limited ability to perform mental tasks (*χ*^*2*^ = 5.020; *df* = 1; *P* = 0.025), and duration of disease since diagnosis (*χ*^*2*^ = 10.758; *df* = 1; *P* = 0.001) were statistically significant.

**Table 2:** Employment reduction among FCGs by cancer survivors’ characteristics in post treatment Phase

Variables	Reduced = 1	Sustained = 0	*χ*^*2*^	*P- value*
	*N*	*N*		
Residence area			52.542	< 0.001
Urban	65	290		
Rural	293	432		
Age group (years)			4.819	0.186
≤ 40	8	33		
40–55	102	179		
56–65	119	240		
> 65	129	270		
Gender			6.075	0.014
Male	171	288		
Female	187	434		
Marital status			6.002	0.014
Married	333	637		
Others	25	85		
Educational level			41.948	< 0.001
Uneducated	60	114		
Elementary school	160	223		
Middle school	113	233		
High school and above	25	152		
Annual household income^a^, Chinese Yuan			22.662	< 0.001
< 5000	73	99		
5000–20,000	143	257		
20,000–50,000	112	234		
> 50,000	29	127		
Cancer site			2.374	0.499
Breast	115	264		
Lung	50	100		
Stomach	90	173		
Colorectal	103	185		
Stage at diagnosis			3.203	0.202
0-I	159	301		
II	112	275		
III-IV	24	46		
Comorbidity			8.356	0.004
Yes	74	205		
No	278	493		
Chemotherapy			10.205	0.001
Yes	272	480		
No	86	242		
Surgery			0.278	0.598
Yes	316	645		
No	42	77		
Radiation therapy			0.063	0.802
Yes	41	79		
No	317	643		
Limited ability to perform physical tasks			49.151	< 0.001
Yes	274	331		
No	14	109		
Limited ability to perform mental tasks			5.020	0.025
Yes	148	194		
No	145	266		
Duration of disease since diagnosis			10.758	0.001
2–3 years	255	441		
4–5 years	103	281		

^a^ 10,000 Chinese Yuan ≈1541 US dollars as of December 31, 2015;

The results of separate multivariable logistic regression models that controlled for patient demographic characteristics are displayed in Table 3. For survivors of stage II cancer (OR = 1.481; 95CI%: 1.083, 2.024), the probability that their FCGs’ employment time would be reduced was higher than that of the comparison group. For survivors who had already undergone chemotherapy treatment (OR = 1.638; 95CI%: 1.202, 2.232) and who suffered from additional chronic diseases (OR = 1.593; 95CI%: 1.148, 2.210), their FCGs were also more likely to make employment reduction decision. Besides, FCGs caring for cancer survivors whose ability to perform physical tasks (OR = 1.742; 95%CI: 3.154, 10.339) was limited, demonstrated a higher likelihood of reducing their working hours. Similarly, FCGs caring for survivors whose ability to perform mental tasks (OR = 1.513; 95CI%: 1.100, 2.081) was limited were also more likely to make work time adjustments to meet caretaking demands.

**Table 3:** Associated factors of employment reduction among FCGs from separate multivariable logistic regression analyses

	Odd Ratio, OR									
	**Model 1**	**Model 2**	**Model 3**^b^	**Model 4**	**Model 5**	**Model 6**	**Model 7**	**Model 8**	**Model 9**	**Model 10**
**Age at time of survey**^**a**^	0.992	0.997	0.998	0.995	0.995	0.992	0.993	0.993	1.003	0.993
**Residence area**										
Urban	Ref									
Rural	2.191***	2.136***	2.314***	2.534***	2.082***	2.259***	2.223***	2.078***	1.965**	2.252***
**Gender**										
Male	Ref									
Female	0.720*	0.704*		0.730*	0.701*	0.722*	0.718*	0.730*	0.821	0.697*
**Marital status**										
Others	Ref									
Married	2.051**	2.176**	2.098**	1.860*	1.994**	2.105**	2.049**	2.052**	1.862	2.229*
**Education**										
Uneducated	Ref									
Elementary school	1.204	1.148	1.276	1.093	1.204	1.220	1.207	1.240	1.119	1.230
Middle school	0.802	0.766	0.896	0.751	0.786	0.810	0.803	0.822	0.626	0.695
High school and above	0.385**	0.351*	0.433**	0.363**	0.384**	0.388**	0.391**	0.395**	0.388*	0.306**
**Annual household income, Chinese Yuan**										
< 5000 CNY	Ref									
5000–19,999 CNY	0.685	0.675	0.683	0.778	0.685	0.693	0.681	0.682	0.776	0.728
20,000–50,000 CNY	0.742	0.744	0.728	0.873	0.692	0.748	0.741	0.734	0.976	0.927
> 50,000 CNY	0.578	0.600	0.569	0.584	0.536*	0.593	0.575	0.573	0.976	0.921
**Comorbidity**										
0		Ref								
≥ 1		1.593**								
**Cancer site**										
Colorectal			Ref							
Breast			1.021							
Lung			0.933							
Stomach			0.992							
**Cancer stage at diagnosis**										
0-I				Ref						
II				1.481*						
III-IV				1.394						
**Chemotherapy**										
No					Ref					
Yes					1.638**					
**Surgery**										
No						Ref				
Yes						0.736				
**Radiation therapy**										
No							Ref			
Yes							1.187			
**Duration of disease since diagnosis**										
2–3 years								Ref		
4–5 years								1.249		
**Limited ability to perform physical tasks**										
No									Ref	
Yes									5.710***	
**Limited ability to perform mental tasks**										
No										Ref
Yes										1.513*

^a^ Age was entered as a continuous variable in the regression model

^b^ The model 3 for cancer site adjusted for residence area, age, marital status, annual household income, and educational level. Models 2, 4–10 adjusted for residence area, age, gender, marital status, annual household income, and educational level

***, **, * indicates *P* < 0.001, *P* < 0.01; *P* < 0.05, respectively

## Discussion

The study demonstrated that the FCGs caring cancer survivors in China made a variety of employment changes after assuming their caregiving responsibilities: 24.6% of FCGs reduced their working hours and 6.4% of FCGs left workforce completely to provide care for the patients (in the post-treatment phase). Our results correspond to de Moor et al.’s study in which approximately 25% of cancer survivors reported that their informal caregivers made substantial employment changes for providing care [17]. Clearly, a notable number of FCGs caring for long-term survivors both nationwide and worldwide have experienced significant reduction in work hours. More importantly, we found that the following cancer-related characteristics were associated with a higher risk of reducing or quitting work among FCGs, which may help identify patients and FCGs in need of employment-related supportive services.

Our study found that patients who suffered from multiple chronic diseases were more likely to influence the employment status of their FCGs. Existing research also found that caregiving for survivors with multiple chronic diseases has negative impact on the HRQoL of their caregivers. For example, Kurtz et al. reported that 73.4% of cancer patients with comorbidities negatively affected the HRQoL of their caregivers [28]. Our study contributes to the literature by confirming that accompanying chronic diseases could be an indicator of a higher probability of employment changes among FCGs. Comorbidities might lead to a variety of complications and hardships, such as life-long disability, household productivity loss, and extra days confined to bed due to poor health condition, which, in turn, affects FCGs’ working patterns.

In this study, caring for patients treated with chemotherapy was more likely to adversely affect the employment status of their caregivers. This is consistent with several existing studies [17, 29], all of which found that the probability of altering employment status was higher among FCGs caring for patients who received chemotherapy than among caregivers for patients who did not receive this type of treatment. Compared to other therapy methods, chemotherapy is regarded as a more traumatizing experience by both patients themselves and their families due to the lengthiness, multiple side effects and difficult recovery period, combined with pain and poor prognosis. In traditional Chinese culture, caregivers usually accompany patients throughout the whole course of hospitalization and stay at the bedside until their loved ones are discharged. Facing substantial care burdens, they may have to modify their work schedule or leave the workforce completely. However, a study conducted in the US showed that although chemotherapy treatment was associated with a decline in caregivers’ involvement in their own daily routines, it was not a highly significant predictor [30]. The discrepancy might be explained by the difference in treatment settings and approaches. In the US, most cancer patients undergo chemotherapy in an outpatient clinic, which can be completed within 3 to 4 h each time [31]. Although some Chinese hospitals have the ability to provide chemotherapy treatment in an outpatient clinic as well, the social health insurance system in China is oriented toward inpatient expenditures rather than outpatient services due to reimbursement rate and ceiling amount schemes. As a result, Chinese cancer patients prefer receiving chemotherapy treatment in a hospital setting [32]. Given the existence of contradictory findings and the specificities of Chinese cultural context, more research is needed to determine whether caring for a patient receiving chemotherapy is indeed an important factor aggravating the risk of employment reduction or loss among FCGs.

FCGs caring for cancer patients with limited ability to perform physical and mental tasks were also more likely to experience worktime-related consequences. This finding corresponds with the results of most other existing studies [15, 17, 33–35]. Ability to perform physical or mental tasks is a basic indicator for measuring physical and mental health of patients. Functional limitation of patients increases time and care demands for FCGs, thus forcing FCGs to sacrifice work hours for providing more care. This problem can even be more acute in the contexts where the cancer patient was responsible for most of daily chores and/or other domestic duties, while the tasks that the patient previously performed may have been transferred to their FCGs upon diagnosis. To maintain the normalcy of family life, FGGs may be obligated to dedicate a portion of their working hours for performing these tasks and other family duties in addition to caregiving.

Finally, the observation that there is a strong relationship between employment time reduction and caring for patients diagnosed with the stage II was an unexpected finding. This did not concur with previous studies as existing studies almost exclusively report that patients diagnosed with advanced-stage cancers tend to place the heaviest burden on the work schedules of FCGs. For example, a recent study showed that there is a strong link between cancer patients who had been diagnosed at an advanced stage and employment losses among FCGs caring for these patients [36]. This discrepancy may be partly explained by the characteristics of participants included in this study’s sample. Cancer survivors diagnosed at later (III-IV) stages of cancer accounted for a relatively small proportion in our sample, and the majority of survey participants were patients diagnosed with the stage II cancer.

This study assessed the employment changes among FCGs caring for cancer survivors, and identified several disease-related factors that are associated with a higher possibility of leading to employment reduction or loss among FCGs. The results suggest that future interventions should emphasize support strategies specifically targeting FCGs caring for cancer patients with the high-risk characteristics identified in this study. Many other factors not accounted for in this study may also play an important role in the employment changes of FCGs. For example, the existing labor law governing “sick leave” in China does not provide any legitimate job-protection for FCGs [37]. In addition, a relatively small family size in current China may future increase the degree of dependence relationship among family members [20]. These factors are, however, out of scope for this study. Nevertheless, given these contributing factors and the findings of this study, it is possible to argue that FCGs might ultimately become the “hidden victims” of cancer care, and therefore a high priority should be given to develop intervention initiatives targeting these individuals.

### Limitations and future directions

Several limitations of this study need to be mentioned. Firstly, information about demographic characteristics and employment types (e.g. part-time versus full-time) of FCGs was lacking, prohibiting a robust analysis of associations between each occupational type and employment reduction or loss. Future investigations focusing on the FCGs’ perspective might uncover fresh insights into factors affecting their employment time changes. Secondly, reporting bias is inevitable in our study. Patients may over- or under- report the employment changes that their FCGs had suffered due to difference in understanding of working hours, the severity of distress and other factors, such as the type and closeness of familial relationship between patients and the FCGs. However, as the survey was conducted at patients’ homes, in some cases FCGs were present at the time of survey administration and assisted the patients by correcting or adding details about their employment conditions if the patient did not have a clear memory or understanding. FCGs also helped patients fill out the questionnaire (with the patients’ permission) if the patient did not have the ability or energy to participate in the whole process. Those special circumstances helped in reducing the reporting bias of cancer survivors to some extent. More research employing indicators with higher accuracy and validity will be of value to better understand the mechanisms behind employment time changes and caring for cancer survivors.

All in all, the cross-sectional design of this study displays only a snapshot view. With cancer gradually becoming a chronic disease, the experience of cancer survivors and their FCGs needs to be addressed with due seriousness. Future research may benefit from larger comparative studies with longitudinal design to assess and compare whether and how employment conditions of FCGs and their patients evolve throughout different stages of cancer. A longitudinal study of this kind is planned as the next stage of this research.

## Conclusions

As can be seen, Chinese FCGs tend to sacrifice or neglect their employment needs when faced with the need to care for their loved ones struck with cancer. This, in turn, may have a range of negative consequences for FCGs such as an interrupted working schedule, a lower household income, or other hardships for the family. Unfortunately, support for FCGs caring for cancer survivors is rarely offered in China. The findings of this research indicate that there is a need to develop interventions tailored to the needs of FCGs to help them efficiently reconcile cancer caregiving activities with employment demands. The findings of this study can also be used to identify individuals who are at the higher risk of employment reduction or loss by looking at the cancer-related characteristics that this study found to be associated with higher probability of employment change.

Healthcare authorities can more effectively identify FCGs with higher risk of negative employment changes by focusing on cancer-related characteristics of their care recipients, such as cancer stage, treatment method, and patients’ mental or physical abilities. It is then important to evaluate and understanding the life changes that FCGs had to go through after a loved one’s cancer diagnosis and treatment, in order to identify the difficulties and demands of FCGs and their families. Finally, patients’ long-term home care and FCGs’ well-being might benefit from developing and implementing a series of appropriate and effective social support programs for cancer care, such as provision of caregiver facilities, support of secondary caregivers, economic subsidy for those affected, improvement of workspace flexibility measures, and legal protection of caregivers’ well-being and jobs.

## Supplementary Information

**Additional file 1:** Related items from the questionnaire of the survey “Your Experience with Cancer in China”

**Additional file 2: Figure 1.** Geographic distribution of sampling counties and districts

## Data Availability

The datasets used and/or analysed during the current study are available from the corresponding author on reasonable request.

## Abbreviations

FCGs: Family caregivers
HRQoL: Health-related quality of life
UEBMI: The urban employee basic medical insurance
URBMI: The urban resident basic medical insurance
RNCMS: The rural new cooperative medical scheme
