# Supplementary material for: Employment changes among Chinese family caregivers of long-term cancer survivors
Source: BMC Public Health. 2020 Nov 25;20:1787. doi: 10.1186/s12889-020-09922-9 (PMC7690119; doi:10.1186/s12889-020-09922-9)
Supplement: Supplementary file 1 — Additional file 1: Related items from the questionnaire of the survey “Your Experience with Cancer in China” [file 12889_2020_9922_MOESM1_ESM.docx]

**Additional file 1 Questionnaire for the survey “Your Experience with Cancer in China”**

(* This survey contains three sections. Section I consists of basic demographic characteristics, and general past medical treatment experience of patients; Section II consists of seeking treatment behavior of patients before and after diagnosis; and Section III includes whole medical trajectory, present treatment status and life condition of patients. Contents about the cancer-related characteristics of patients and employment conditions of family caregivers were included in the Section III, thus, we listed related items from the Section III as the Additional file 2 for help.)

**SECTION III**

1. By the time you were diagnosed cancer, do you have any chronic diseases?
   1. Diabetes
   2. Cardiovascular Diseases (i.e.: hypertension, heart disease)
   3. Respiratory Diseases
   4. Others: ___________
   5. None
2. After you were diagnosed cancer, what cancer treatments did you receive? (check all that apply)
   1. Surgery
   2. Chemotherapy
   3. Radiation Therapy
   4. Hormonal Therapy
   5. Targeted Therapy
   6. Chinese herbal medicine
   7. Others: _________________
3. What cancer treatments are you planning to receive?
   1. None
   2. Surgery
   3. Chemotherapy
   4. Radiation Therapy
   5. Hormonal Therapy
   6. Targeted Therapy
   7. Chinese herbal medicine
   8. Others: _________________
   9. Follow physician’s advices
4. When did you receive your most recent cancer treatment?
   1. Within 1 year
   2. 1 year ago but less than 3 years
   3. 3 years ago but less than 5 years
   4. 5 years ago but less than 10 years
   5. 10 years ago but less than 20 years
   6. More than 20 years
   7. I have never received any kind of cancer treatment
5. Did a doctor or other health professional ever tell you that your cancer had come back?
   1. Yes
   2. No

***If you were retired or unemployed by the time you were diagnosed cancer, please skip Question 6.***

1. At any time since you first diagnosed cancer, did you make any changes for your work schedule? If so, could you briefly talk about it?

­­­­­­_________________________________________________________________________

1. Because of your cancer, its treatment, or the lasting effects of that treatment, did you change your retirement schedule?
   1. Yes, I retired earlier than the time I have planned
   2. No
   3. Yes, I retired later than the time I have planned
   4. Not applicable (I was retired by the time I was first diagnosed)
2. Because of your cancer, its treatment, or the lasting effects of that treatment, did any of your family caregivers change their retirement schedules?
   1. Yes, they retired earlier than the time they have planned
   2. No
   3. Yes, they retired later than the time they have planned
   4. Not applicable (they were retired by the time I was first diagnosed)
3. **At any time since your first cancer diagnosis, did you get another job because of your cancer, its treatment, or the lasting effects of the treatment?**
   1. **Yes**
   2. **No**
   3. Not applicable (I was retired by the time I was first diagnosed)
4. **At any time since your first cancer diagnosis, did** any of your family caregivers **get another job because of your cancer, its treatment, or the lasting effects of the treatment?**
   1. **Yes**
   2. **No**
   3. Not applicable (I was retired by the time I was first diagnosed)
5. Throughout your cancer treatment, did you shorten your paid working hours because of your cancer, its treatment or the lasting effects of the treatment?
   1. No. I extended my working hours.
   2. Not so much, I kept my regular working hours.
   3. Yes, I reduced less than half of my working hours.
   4. Yes, I reduced half of my working hours.
   5. Yes, I reduced more than a half of my working hours.
   6. Yes, I stopped working.
   7. Not applicable (I was retired by the time I was first diagnosed)

11a. If your working hours have been shortened, throughout your cancer treatment, your working schedule reduced _____ days.

1. **Throughout your cancer treatment, did any of your family caregivers shorten their paid working hours because of your cancer diagnosis, its treatment or the after effects of the treatment?**
   1. **No. They extended their working hours.**
   2. **Not so much, they kept their regular working hours.**
   3. **Yes, they reduced less than half of their working hours.**
   4. **Yes, they reduced half of their working hours.**
   5. **Yes, they reduced more than a half of their working hours.**
   6. **Yes, they stopped working.**
   7. **Not applicable (They were retired by the time I was first diagnosed)**

12a. If their working hours have been shortened, their working schedule reduced _____ days.

***The following questions are designed for patients who have completed cancer treatment (surgery, chemotherapy and radiation therapy) more than 1 year.***

1. After 1 year completed the cancer treatment, did you shorten your paid working hours because of your cancer, its treatment or the lasting effects of the treatment?
   1. No. I extended my working hours.
   2. Not so much, I kept my regular working hours.
   3. Yes, I reduced less than half of my working hours.
   4. Yes, I reduced half of my working hours.
   5. Yes, I reduced more than a half of my working hours.
   6. Yes, I stopped working.
   7. Not applicable (I was retired by the time I was first diagnosed)

13a. If your working hours have been shortened, your working schedule reduced _____ days.

1. **After having completed your treatment for a period of at least one year, did any of your family caregivers shorten their paid working hours because of your cancer diagnosis, its treatment or the lasting effects of the treatment?**
   1. **No. They extended their working hours.**
   2. **Not so much, they kept their regular working hours.**
   3. **Yes, they reduced less than half of their working hours.**
   4. **Yes, they reduced half of their working hours.**
   5. **Yes, they reduced more than a half of their working hours.**
   6. **Yes, they stopped working.**
   7. **Not applicable (They were retired by the time I was first diagnosed)**

14a. If their working hours have been shortened, their working schedule reduced _____ days.

1. After 1 year completed the cancer treatment, was your income reduced because of cancer, its treatment or the lasting effects of the treatment?
   1. No. My income has increased.
   2. Not so much, my income was not affected by the cancer.
   3. Yes, my income has decreased less than a half of my original income
   4. Yes, my income has decreased a half of my original income
   5. Yes, my income has decreased more than a half of my original income
   6. Yes, I no longer have any income
2. After 1 year completed the cancer treatment, was any of your family caregivers’ income reduced because of cancer, its treatment or the lasting effects of the treatment?
   1. No. the income has increased.
   2. Not so much, the income was not affected by the cancer.
   3. Yes, the income has decreased less than a half of the original income
   4. Yes, the income has decreased a half of the original income
   5. Yes, the income has decreased more than a half of the original income
   6. Yes, they no longer have any income

1. Did you ever feel that your cancer, its treatment, or the lasting effects of that treatment interfered with your ability to perform any physical tasks required by your job?
   1. Yes
   2. No
   3. No, I don’t need to perform any physical tasks during work.
   4. Not applicable (I was retired by the time I was first diagnosed)
2. At any time after you have completed your cancer treatment, have you felt any level of memory loss during work?
   1. Yes
   2. No
   3. No, I don’t need to perform any mental tasks during work.
   4. Not applicable (I was retired by the time I was first diagnosed)
3. Did you ever feel that, because of your cancer, its treatment, or the lasting effects of that treatment, your productivity at work are different from before??
   1. Increased
   2. Decreased
   3. About the same.
   4. Not applicable (I was retired by the time I was first diagnosed)

…

**Thank you so much for completing our survey.**
