# Supplementary material for: Employment changes among Chinese family caregivers of long-term cancer survivors
Source: BMC Public Health. 2020 Nov 25;20:1787. doi: 10.1186/s12889-020-09922-9 (PMC7690119; doi:10.1186/s12889-020-09922-9)
Supplement: Supplementary file 2 — Additional file 2: Figure 1. Geographic distribution of sampling counties and districts [file 12889_2020_9922_MOESM2_ESM.docx]

**Additional file 2** **Geographic distribution of sampling counties and districts**


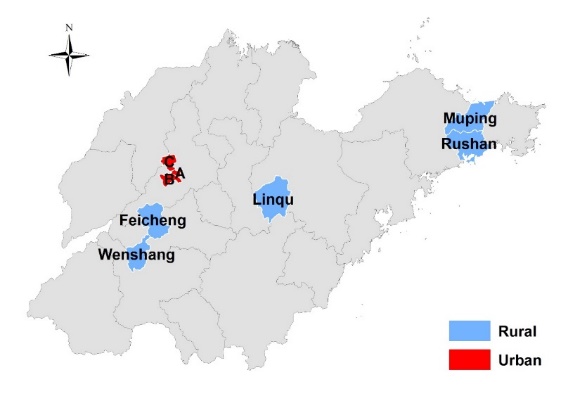


**Additional figure 1 Geographic distribution of sampling counties and districts**

* Counties and districts are at the same level in the Chinese administrative division system. A represents the Lixia District, B represents the Shizhong District, and C represents Tianqiao District of Jinan (the Capital city of Shandong Province) in the map. The map was mapped using ArcGIS 10.1(ESRI, RedLands, USA).
